# Care of Children’s Teeth

**Published:** 1884-07

**Authors:** Minnie F. Mosher


### Care of Children's Teeth.

BY MINNIE F. MOSHER, D. D. S.

Since diseased teeth produce so many evil effects on the general health, if the child is the father of the man, it seems as if dentists ought to turn their attention, with more care than heretofore, to the teeth of children. Parents should be brought face to face with their carelessness in this matter—as an offence against civilization in its high sense. If mankind cannot be perfect so long as the laws of Nature are not obeyed, it behooves every one to strive to bring about such obedience.

Passing over the sinning against Nature, which brings into the world children with defective organizations, inclusive of congenital malformation of the teeth, obedience to Nature's laws of cleanliness and self-preservation, and man's art in atoning for past disobedience, might do much to favor improvement in the case of the teeth.

The rate of advancement in this matter lies in the influence and zeal of the dentist.

Let him insist on having the children placed in his care, as in the case of the family physician; but let him consider a thorough knowledge of the proper treatment of children's mouths, as essential for the foundation in the practice of his profession, as the Greek alphabet is to the student who would read Homer's Iliad in the original text. Let him do none but conscientious work. He should insist that children should be brought to him for an examination of their teeth as often as once in three months until the completion of the second dentition; then once in six months ought to be often enough. A record should be kept of the time at which each child presents itself, and delinquents should promptly be notified. A dentist should do his duty with pen, as well as with tongue and fingers. True the masses do not read the dental journals, but they do read the newspaper; why not insert an occasional article of plain information concerning children's teeth in a daily paper?—the evening paper is preferable; then people have more time to read.

Let the dentist give such instructions as these to parents, enforcing them upon the children whenever an opportunity offers.

The teeth of children should be watched as well as the general health. The child should be instructed in the various precautions against decay of the teeth. It should be taught that the teeth are for the purpose of mastication; not to be used to crack nuts, or to hold nails, or to bite off wires, or to pull out bent pins. It should also be taught to rinse the mouth after each meal, then as it grows older, to use a toothpick, floss silk, and a brush, at least twice a day, after each meal would be better. Many a child is allowed to retain on the teeth substances, which, if it were to put into them its hands instead of its mouth, would nauseate it, both on account of the odor and the general filthiness.

Hygienic precautions should be taken ; the bath frequently used ; the child, properly dressed, should be allowed plenty of out-of-door exercise ; it should have plenty of sleep in a well ventilated room. The habit of breathing through the mouth should be guarded against, by cautions during the day, at night by requiring the child to persistently close the mouth upon going to bed ; in cases where it is necessary, bandages should be applied to keep the mouth closed.

Now the breathing vitiates the secretions by too rapid evaporation from the mucous membrane ; it brings about an abnormal temperature ; it allows the atmosphere to enter the mouth and fauces without being first purified by the cilia of the nostrils, thereby allowing agents which further decay, to be brought in contact with the teeth ; it also induces irregularity of the teeth ; the incisors and cuspids, lacking their natural antagonists, change their positions, usually protruding. Sucking the thumb or keeping the fingers continually in the mouth leads to irregularity of the teeth, and the habit should be broken up, forcibly, if need be. The habit of chewing gum induces a morbid flow of saliva, and has a ruinous effect upon teeth which have been filled.

The nutrition of the child should be well considered ; food should be given which contains with other nutrient material, a sufficient quantity of calcareous matter, especially during the course of various diseases common to children.

Fruit, good beef, and mutton, milk, wheaten flour, oatmeal, and vegetables, when the child will take them, should be given. Sweets may be given a child in reasonable quantites ; sugar contains much nutriment ; but sweets should be chosen with regard to their purity of composition ; and the child should be instructed not to crush such confections between the teeth, but to let them soften in the mouth ; it is true that the use of the teeth in chewing tough substances is beneficial, but hard substances often injure the enamel. After eating sweets a child should rinse the mouth with water ; it lessens the risk of the sugar being converted into acid in the places on the teeth predisposed by formation to decay. Hot or cold substances, following each other so as to cause a sudden change of temperature, and acids which cause the affection known as "setting the teeth on edge," should be avoided. The dentist should gain the confidence of the children brought to him; and the influence of a well-kept office, fresh towels, and personal nicety will be felt by a child full as soon as by an adult. Parents should be urged not to use the dentist as a bugbear, with threats of which to subdue a refractory mood; the child should be taught to regard a visit to the dentist as a commonplace matter: on a par with washing its face, or eating a piece of bread and butter. If a child asks about the pain of an impending operation, tell it the truth, promise to be careful, and usually the bravery it will display, will compare favorably with the fortitude of his elders. Deal gently with the mouth—make operations as short as possible; better take two days for a case of cleaning, or excavate a cavity one day, and fill the next—protecting the cavity from outside influences in meantime, than exhaust the patient. Never lose patience with a child, nor become fussy or undecided. Take into consideration your own and the patient's mood. If you are to work for a stranger, inquire into its history, learn its diathesis and mode of living. In an examination of the mouth use litmus paper and a pocket lens, also a fine exploring instrument, and floss silk. Do not allow a child to witness unpleasant operations, or to hear them described; uneducated people especially, make much ado about simple operations, and children gain from their conversation, wrong ideas. As soon as the form of deposit, known as green stain, appears on the teeth, it should be removed by means of proper appliances; and the surfaces of the teeth polished. The deciduous teeth should be preserved as well as possible, until nature's own time for removing them. Care should be taken in the removal of the deciduous teeth, that they may be neither extracted too soon, thus allowing the jaw to contract, and the permanent teeth to be crowded, nor left too long in the jaw, thus causing irregularity of the permanent teeth, or as in some cases, altogether preventing their eruption. It is usually best not to fill the deciduous teeth with gold; tin may be used, or oxy-phosphate, or Hill's stopping; for a mere temporary filling gutta percha may be used.

The gums about the teeth can be kept in a healthy condition by cleanliness, constitutional treatment, and the application of an astringent in the shape of a crystal of rock salt.

A child should be instructed to favor the first molars till they become matured; these teeth are frequently defective, especially if proper food is not given to satisfy the wants of the growing body.

As soon as is practicable any fissures in the enamel, covering the masticating surfaces of the teeth, should be properly cut and filled.

Care should be taken about putting gold into a maturing tooth. The irritation incidental to the proper condensation of the gold in some teeth would injure the pulp, and render it susceptible to decay producing agents, or destroy it entirely. For this reason the judgment of the operator should determine whether a maturing tooth, which has been broken, and is unsightly, should be built down; if it is left to mature first, the ragged edges should be made smooth. Tin can be used on the masticating surfaces; on non-masticating surfaces fillings of Hall's stopping or oxy-phosphate. They will often last for years. In children's teeth the pulp canal is often large, especi ally in the anterior teeth; in excavating cavities in such teeth, care should be taken not to cut into the pulp canal.

Small cavities should be filled as soon as discovered. Teeth in which only a small part of the substance is devitalized are of more value in the mouth than those which have become mere shells, because the cavities in them were left uncared for till they should become "large enough to fill." Appliances for irregularities, such as straightening the teeth, expanding the jaw, or drawing the jaw back into a position in which the teeth may antagonize, should be used before the tissues become mature and unyielding—the change is then more easily accomplished.

The writer offers the thoughts in this paper, not as covering the whole ground of the subject under discussion, but as reaching out toward the proper prophylactics for diseases of the teeth.

